# Supplementary material for: Genome-Wide Identification and Analysis of Family Members with Juvenile Hormone Binding Protein Domains in Spodoptera frugiperda
Source: Insects. 2024 Jul 28;15(8):573. doi: 10.3390/insects15080573 (PMC11354444; doi:10.3390/insects15080573)
Supplement: Supplementary file 1 [file insects-15-00573-s001.zip › Table S1 Physical and chemical properties and subcellular localization of JHBP gene family in S. frugiperda.pdf]

Table S1: Physical and chemical properties and subcellular localization of *JHBP* gene family in *S. frugiperda*

Gene name	Gene ID	Amount of amino acid	Molecular weight	Isoelectric point	Coefficient of instabiliy	Grand average hydrophobiciy	Subcellular localization
SfJHBP1	118279107	252	28925.16	6.19	16.63	-0.283	extr
SfJHBP2	118279117	252	28939.19	6.19	17.39	-0.282	extr
SfJHBP3	118263051	226	24971.7	5	31.9	0.274	extr
SfJHBP4	118263052	226	25099.43	4.44	30.69	0.223	extr
SfJHBP5	118262973	237	26643.57	4.76	39.15	0.144	plas
SfJHBP6	118263150	242	26872.87	4.29	43.37	0.277	extr
SfJHBP7	118263164	242	26892.87	4.55	38.59	0.245	extr
SfJHBP8	118263085	257	28176.62	4.8	32.24	0.25	E.R.
SfJHBP9	118263053	226	24971.7	5	31.9	0.274	extr
SfJHBP10	118263054	226	25099.43	4.44	30.69	0.223	extr
SfJHBP11	118262989	237	26643.57	4.76	39.15	0.144	plas
SfJHBP12	118263148	242	26872.87	4.29	43.37	0.277	extr
SfJHBP13	118263162	242	26892.87	4.55	38.59	0.245	extr
SfJHBP14	118263086	257	28176.62	4.8	32.24	0.25	E.R.
SfJHBP15	118263038	259	28611.21	6.9	29.11	0.092	extr
SfJHBP16	118262843	247	27228.34	5.23	26.56	-0.006	extr
SfJHBP17	118263018	262	29215.66	5.79	57.64	-0.096	extr
SfJHBP18	118263001	250	27307.94	9.2	32.15	0.064	extr
SfJHBP19	118262220	196	21978.5	8.92	39.26	-0.195	cyto
SfJHBP20	118262190	282	31315.4	8.38	20.94	-0.022	cyto
SfJHBP21	118262188	457	51306.75	4.87	23.59	-0.061	E.R.
SfJHBP22	118262185	460	50909.32	4.87	32.45	-0.069	E.R.
SfJHBP23	118262192	247	27668.5	5.72	37.59	-0.204	extr
SfJHBP24	118262222	189	21806.51	6.97	18.73	-0.478	cyto
SfJHBP25	118262191	282	31188.21	8.08	21.23	-0.009	cyto
SfJHBP26	118262187	457	51294.69	4.87	23.77	-0.072	E.R.
SfJHBP27	118262186	459	50775.1	4.83	32.62	-0.081	E.R.
SfJHBP28	118263049	247	27668.5	5.72	37.59	-0.204	extr
SfJHBP29	118262276	251	28927.27	9.09	29.98	-0.253	extr
SfJHBP30	118262682	274	30621.78	4.7	29.8	0.057	extr
SfJHBP31	118262685	265	29784.61	5.26	46.08	0.14	extr
SfJHBP32	118262690	255	27733.93	6.19	18.16	0.129	extr
SfJHBP33	118262693	245	27044.48	8.94	25.99	0.069	extr
SfJHBP34	118262681	274	30634.69	4.67	30.75	-0.008	extr
SfJHBP35	118262686	265	29842.65	5.16	44.66	0.112	extr
SfJHBP36	118262689	255	27749.93	6.19	18.92	0.119	extr
SfJHBP37	118262705	243	26771.08	8.74	25.97	0.066	extr
SfJHBP38	118262248	234	26593.43	4.94	37.19	-0.071	extr

SfJHBP39	118263082	256	27725.38	5.09	35.26	0.432	extr
SfJHBP40	118263822	249	28063.43	5.72	36	-0.165	extr
SfJHBP41	118263879	249	28048.42	5.71	34.63	-0.173	extr
SfJHBP42	118265222	295	33162.56	8.84	39.27	-0.155	mito
SfJHBP43	118275183	249	28140.81	8.39	34.68	-0.1	extr
SfJHBP44	118275983	544	60148.08	8.5	27.03	0.059	extr
SfJHBP45	118276116	235	26130.34	8.19	30.38	-0.032	extr
SfJHBP46	118276477	239	26795.04	5.76	22.56	-0.093	extr
SfJHBP47	118276492	242	26690.09	6	27.69	0.017	extr
SfJHBP48	118276310	239	26611.01	8.53	35.93	-0.069	extr
SfJHBP49	118276311	196	21889.65	9.03	25.88	-0.075	extr
SfJHBP50	118275991	487	54131.44	6.99	26.7	0.033	E.R.
SfJHBP51	118276282	249	27907.15	5.12	26.79	-0.085	extr
SfJHBP52	118276515	241	27201.6	6.59	36.35	-0.116	extr
SfJHBP53	118276493	235	27235.11	5.17	35.47	-0.233	extr
SfJHBP54	118276335	235	27615.88	8.44	25.15	-0.329	mito
SfJHBP55	118275992	221	25687.81	6.98	40.48	-0.269	extr
SfJHBP56	118276338	234	26839.57	8	46.92	-0.496	extr
SfJHBP57	118276339	238	27409.58	8.34	33.7	-0.295	extr
SfJHBP58	118276340	236	26668.63	5.56	20.18	-0.263	extr
SfJHBP59	118276429	235	26530.42	5.76	21.22	-0.302	extr
SfJHBP60	118276399	244	27059.15	5.39	23.36	-0.085	extr
SfJHBP61	118276400	244	26939.28	5.37	30.42	0.161	extr
SfJHBP62	118276102	279	31723.22	7.66	51.25	-0.286	extr
SfJHBP63	118276000	505	56247.99	8.26	27.3	0.032	E.R.
SfJHBP64	118276414	243	27623.86	5.39	30.03	-0.091	extr
SfJHBP65	118276352	259	29487.18	6.2	27.83	-0.184	extr
SfJHBP66	118276283	261	29064.59	4.88	21.13	0.074	extr
SfJHBP67	118276317	261	29952.39	5.05	43.56	-0.012	extr
SfJHBP68	118276328	251	28374.83	7.52	26.67	-0.016	extr
SfJHBP69	118276479	251	28543.22	6.9	22.84	0.017	extr
SfJHBP70	118276090	244	27339.98	8.21	20.86	-0.015	extr
SfJHBP71	118276456	255	28818.4	8.6	31.93	-0.163	extr
SfJHBP72	118276100	240	26633.9	8.51	41.51	-0.058	extr
SfJHBP73	118276379	263	30815.61	5.53	32.11	-0.182	extr
SfJHBP74	118276378	263	30815.61	5.53	32.11	-0.182	extr
SfJHBP75	118276823	212	23711.35	5.47	23.5	-0.132	extr
SfJHBP76	118278230	196	22116.89	9.25	23.91	-0.056	extr

Note: cyto: cytoplasm. mito: mitochondria. extra: extracellular. E.R: Endoplasmic Reticulum.
