# Supplementary material for: Genome-Wide Identification and Analysis of Family Members with Juvenile Hormone Binding Protein Domains in Spodoptera frugiperda
Source: Insects. 2024 Jul 28;15(8):573. doi: 10.3390/insects15080573 (PMC11354444; doi:10.3390/insects15080573)
Supplement: Supplementary file 1 [file insects-15-00573-s001.zip › Table S2 Secondary structure analysis of the JHBP protein of S. frugiperda.pdf]

Table S2: Secondary structure analysis of the JHBP protein of *S. frugiperda*

Gene name	Alpha helix	Beta turn	Random coil	Extended strand	Signal peptide position	cleavage site
SfJHBP1	92(36.51%)	9(3.57%)	90(35.71%)	61(24.21%)	1-19	19-20
SfJHBP2	95(37.70%)	14(5.56%)	85(33.73%)	58(23.02%)	1-19	19-20
SfJHBP3	105(46.46%)	9(3.98%)	62(27.43%)	50(22.12%)	1-16	16-17
SfJHBP4	107(47.35%)	7(3.10%)	61(26.99%)	51(22.57%)	1-16	16-17
SfJHBP5	108(45.57%)	12(5.06%)	55(23.21%)	62(26.16%)	1-19	19-20
SfJHBP6	129(53.31%)	7(2.89%)	66(27.27%)	40(16.53%)	1-30	30-31
SfJHBP7	113(46.69%)	10(4.13%)	68(28.10%)	51(21.07%)	1-30	30-31
SfJHBP8	111(43.19%)	12(4.67%)	74(28.79%)	60(23.35%)	1-18	18-19
SfJHBP9	105(46.46%)	9(3.98%)	62(27.43%)	50(22.12%)	1-16	16-17
SfJHBP10	107(47.35%)	7(3.10%)	61(26.99%)	51(22.57%)	1-16	16-17
SfJHBP11	108(45.57%)	12(5.06%)	55(23.21%)	62(26.16%)	1-19	19-20
SfJHBP12	129(53.31%)	7(2.89%)	66(27.27%)	40(16.53%)	1-30	30-31
SfJHBP13	113(46.69%)	10(4.13%)	68(28.10%)	51(21.07%)	1-30	30-31
SfJHBP14	111(43.19%)	12(4.67%)	74(28.79%)	60(23.35%)	1-18	18-19
SfJHBP15	90(34.75%)	13(5.02%)	84(32.43%)	72(27.80%)	1-25	25-26
SfJHBP16	83(33.60%)	14(5.67%)	82(33.20%)	68(27.53%)	1-15	15-20
SfJHBP17	105(40.08%)	12(4.58%)	85(32.44%)	60(22.90%)	1-18	18-19
SfJHBP18	91(36.40%)	11(4.40%)	83(33.20%)	65(26.00%)	1-16	16-17
SfJHBP19	66(33.67%)	12(6.12%)	63(32.14%)	55(28.06%)	1-24	24-25
SfJHBP20	104(36.88%)	15(5.32%)	88(31.21%)	75(26.60%)	1-54	54-55
SfJHBP21	238(52.08%)	27(5.91%)	115(25.16%)	77(16.85%)	1-17	17-18
SfJHBP22	227(49.35%)	24(5.22%)	127(27.61%)	82(17.83%)	1-19	19-20
SfJHBP23	88(35.63%)	10(4.05%)	86(34.82%)	63(25.51%)	1-18	18-19
SfJHBP24	64(33.86%)	16(8.47%)	66(34.92%)	43(22.75%)	1-12	12-13
SfJHBP25	107(37.94%)	14(4.96%)	92(32.62%)	69(24.47%)	1-54	54-55
SfJHBP26	240(52.52%)	23(5.03%)	121(26.48%)	73(15.97%)	1-17	17-18
SfJHBP27	228(49.67%)	24(5.23%)	132(28.76%)	75(16.34%)	1-18	18-19
SfJHBP28	88(35.63%)	10(4.05%)	86(34.82%)	63(25.51%)	1-18	18-19
SfJHBP29	121(48.21%)	13(5.18%)	65(25.90%)	52(20.72%)	1-23	23-24
SfJHBP30	124(45.26%)	13(4.74%)	81(29.56%)	56(20.44%)	1-17	17-18
SfJHBP31	93(35.09%)	7(2.64%)	111(41.89%)	54(20.38%)	1-19	19-20
SfJHBP32	102(40.00%)	8(3.14%)	106(41.57%)	39(15.29%)	1-16	16-17
SfJHBP33	100(40.82%)	10(4.08%)	75(30.61%)	60(24.49%)	1-21	21-22
SfJHBP34	137(50.00%)	10(3.65%)	68(24.82%)	59(21.53%)	1-17	17-18
SfJHBP35	84(31.70%)	12(4.53%)	110(41.51%)	59(22.26%)	1-19	19-20

SfJHBP36	102(40.00%)	8(3.14%)	106(41.57%)	39(15.29%)	1-16	16-17
SfJHBP37	89(36.63%)	12(4.94%)	69(28.40%)	73(30.04%)	1-21	21-22
SfJHBP38	108(46.15%)	9(3.85%)	71(30.34%)	46(19.66%)	1-20	20-21
SfJHBP39	143(55.86%)	10(3.91%)	63(24.61%)	40(15.62%)	1-15	15-16
SfJHBP40	97(38.96%)	15(6.02%)	78(31.33%)	59(23.69%)	1-18	18-19
SfJHBP41	88(35.34%)	14(5.62%)	77(30.92%)	70(28.11%)	1-18	18-19
SfJHBP42	94(31.86%)	15(5.08%)	122(41.36%)	64(21.69%)	1-18	18-19
SfJHBP43	85(34.14%)	14(5.62%)	79(31.73%)	71(28.51%)	1-17	17-18
SfJHBP44	236(43.38%)	39(7.17%)	135(24.82%)	134(24.63)	1-18	18-19
SfJHBP45	78(33.19%)	10(4.26%)	89(37.87%)	104(19.01%)	1-17	17-18
SfJHBP46	74(30.96%)	9(3.77%)	87(36.40%)	69(28.87%)	1-22	22-23
SfJHBP47	86(35.54%)	12(4.96%)	80(33.06%)	64(26.45%)	1-19	19-20
SfJHBP48	93(38.91%)	12(5.02%)	83(34.73%)	51(21.34%)	1-19	19-20
SfJHBP49	60(30.61%)	10(5.10%)	70(35.71%)	56(28.57%)	1-19	19-20
SfJHBP50	193(39.63%)	25(5.13%)	146(29.98%)	123(25.26%)	1-19	19-20
SfJHBP51	98(39.36%)	10(4.02%)	82(32.93%)	59(23.69%)	1-27	27-28
SfJHBP52	89(36.93%)	12(4.98%)	76(31.54%)	64(26.56%)	1-18	18-19
SfJHBP53	92(39.15%)	8(3.40%)	73(31.06%)	62(26.38%)	1-18	18-19
SfJHBP54	96(40.85%)	12(5.11%)	65(27.66%)	62(26.38%)	1-19	19-20
SfJHBP55	71(32.13%)	12(5.43%)	76(34.39%)	62(28.05%)	1-19	19-20
SfJHBP56	85(36.32%)	17(7.26%)	71(30.34%)	61(26.07%)	1-25	25-26
SfJHBP57	76(31.93%)	17(7.14%)	75(31.51%)	70(29.41%)	1-19	19-20
SfJHBP58	85(36.02%)	13(5.51%)	73(30.93%)	65(27.54%)	1-20	20-21
SfJHBP59	93(39.57%)	16(6.81%)	69(29.36%)	57(24.26%)	1-20	20-21
SfJHBP60	107(43.85%)	12(4.92%)	77(31.56%)	48(19.67%)	1-18	18-19
SfJHBP61	103(42.21%)	10(4.10%)	90(36.89%)	41(16.80%)	1-18	18-19
SfJHBP62	77(27.60%)	25(8.96%)	106(37.99%)	71(25.45%)	1-24	24-25
SfJHBP63	209(41.39%)	35(6.93%)	161(31.88%)	100(19.80%)	1-19	19-20
SfJHBP64	80(32.92%)	12(4.94%)	87(35.80%)	64(26.34%)	1-17	17-18
SfJHBP65	110(42.47%)	16(6.18%)	73(28.19%)	60(23.17%)	1-17	17-18
SfJHBP66	104(39.85%)	23(8.81%)	70(26.82%)	64(24.52%)	1-19	19-20
SfJHBP67	94(36.02%)	17(6.51%)	78(29.89%)	72(27.59%)	1-21	21-22
SfJHBP68	83(33.07%)	12(4.78%)	88(35.06%)	68(27.09%)	1-24	24-25
SfJHBP69	85(33.86%)	14(5.58%)	86(34.26%)	66(26.29%)	1-24	24-25
SfJHBP70	157(28.70%)	25(4.57%)	265(48.45%)	100(18.28%)	1-22	22-23
SfJHBP71	95(37.25%)	18(7.06%)	78(30.59%)	64(25.10%)	1-17	17-18
SfJHBP72	98(40.83%)	17(7.08%)	79(32.92%)	46(19.17%)	1-21	21-22
SfJHBP73	84(31.94%)	12(4.56%)	91(34.60%)	76(28.90%)	1-26	26-27
SfJHBP74	84(31.94%)	12(4.56%)	91(34.60%)	76(28.90%)	1-26	26-27
SfJHBP75	79(37.26%)	13(6.13%)	63(29.72%)	57(26.89%)	1-19	19-20

SfJHBP76	51(26.02%)	17(8.67%)	64(32.65%)	64(32.65%)	1-21	21-22
