# Supplementary material for: Genome-Wide Identification and Analysis of Family Members with Juvenile Hormone Binding Protein Domains in Spodoptera frugiperda
Source: Insects. 2024 Jul 28;15(8):573. doi: 10.3390/insects15080573 (PMC11354444; doi:10.3390/insects15080573)
Supplement: Supplementary file 1 [file insects-15-00573-s001.zip › Table S3 Analysis of gene replication events in SfJHBP family members of S. frugiperda.pdf]

Table S3: Analysis of gene replication events in *SfJHBP* family members of *S. frugiperda*

**Duplicated genes**		**Ka**	**Ks**	**Ka/Ks**	**Divergence time (Million years)**	**Duplicated type**
SfJHBP3	*SfJHBP4*	0.16906	0.57571	0.293656	44.29	Tandem replication
SfJHBP6	*SfJHBP7*	0.294499	0.97957	0.300641	75.35	Tandem replication
SfJHBP9	*SfJHBP10*	0.16906	0.57571	0.293656	44.29	Tandem replication
SfJHBP10	*SfJHBP11*	0.567015	2.17891	0.260228	16.76	Tandem replication
SfJHBP12	*SfJHBP13*	0.294499	0.97957	0.300641	75.35	Tandem replication
SfJHBP15	*SfJHBP16*	0.87467	1.52626	0.573082	11.74	Tandem replication
SfJHBP16	*SfJHBP17*	0.940142	1.43579	0.654792	11.04	Tandem replication
SfJHBP17	*SfJHBP18*	0.802522	1.98734	0.403817	15.29	Tandem replication
SfJHBP21	*SfJHBP22*	0.195319	2.31437	0.084394	17.8	Tandem replication
SfJHBP26	*SfJHBP27*	0.195862	NaN	NaN		Tandem replication
SfJHBP30	*SfJHBP31*	0.837104	NaN	NaN		Tandem replication
SfJHBP31	*SfJHBP32*	0.86638	2.16921	0.399398	16.69	Tandem replication
SfJHBP35	*SfJHBP36*	0.872587	1.97715	0.441337	15.21	Tandem replication
SfJHBP38	*SfJHBP39*	0.837026	1.8058	0.46352	13.89	Tandem replication
SfJHBP45	*SfJHBP46*	0.639797	3.03852	0.210562	23.37	Tandem replication
SfJHBP47	*SfJHBP48*	0.647904	2.30158	0.281504	17.7	Tandem replication
SfJHBP49	*SfJHBP50*	0.021382	0.011	1.943333	84.62	Tandem replication
SfJHBP50	*SfJHBP51*	0.459774	1.83378	0.250725	14.11	Tandem replication
SfJHBP52	*SfJHBP53*	0.602356	NaN	NaN		Tandem replication
SfJHBP54	*SfJHBP55*	0.587137	NaN	NaN		Tandem replication
SfJHBP55	*SfJHBP56*	0.696988	NaN	NaN		Tandem replication
SfJHBP57	*SfJHBP58*	0.681132	2.72178	0.250253	20.94	Tandem replication
SfJHBP58	*SfJHBP59*	0.526699	1.41927	0.371105	10.92	Tandem replication
SfJHBP59	*SfJHBP60*	0.047037	0.04607	1.021062	35.44	Tandem replication
SfJHBP61	*SfJHBP62*	0.125163	0.20305	0.616405	15.62	Tandem replication
SfJHBP62	*SfJHBP623*	0.984574	NaN	NaN		Tandem replication
SfJHBP66	*SfJHBP67*	0.65951	3.429	0.192333	26.38	Tandem replication
SfJHBP67	*SfJHBP68*	0.603022	NaN	NaN		Tandem replication
SfJHBP69	*SfJHBP70*	0.391038	NaN	NaN		Tandem replication
